# Exploring Relationships Between L2 Chinese Character Writing and Reading Acquisition From Embodied Cognitive Perspectives: Evidence From HSK Big Data

**DOI:** 10.3389/fpsyg.2021.779190

**Published:** 2022-02-21

**Authors:** Xingsan Chai, Mingzhu Ma

**Affiliations:** Institute on Educational Policy and Evaluation of International Students, Beijing Language and Culture University, Beijing, China

**Keywords:** Chinese as a second language (CSL) reading, Chinese character writing, Sinosphere, language distance, embodied cognition theory

## Abstract

Chinese characters are central to understanding how learners learn to read a logographic script. However, researchers know little about the role of character writing in reading Chinese as a second language (CSL). Unlike an alphabetic script, a Chinese character symbol transmits semantic information and is a cultural icon bridging embodied experience and text meaning. As a unique embodied practice, writing by hand contributes to cognitive processing in Chinese reading. Therefore, it is essential to clarify how Chinese character writing (bodily activity), language distance (past language usage), and cultural background (bodily coupling with the environment) influence CSL reading proficiency. Based on extant research on L2 reading acquisition and strength of key theoretical perspectives of embodied cognition theory (ECT), this study tested a regression model for CSL reading involving individual-level factors (Chinese character writing proficiency [CCWP]) and group-level predictors (language distance and cultural background). This study collected big data in a sample of 74,362 CSL learners with 67 diverse L1s. Results of hierarchical linear modeling showed a significant effect of CCWP and significant language distance × CCWP interaction effect on reading proficiency; however, cultural background × CCWP interaction effect was not significant. These results conform to the ECT and indicate that bodily activity, past language usage, and cultural background aided reading. CCWP may benefit from withstanding the negative transfer from L1s. Furthermore, CCWP and cultural background are not synergistic predictors of reading. This study may open novel avenues for explorations of CSL reading development.

## Introduction

Reading and writing are inextricably linked. Chinese characters, regarded as most complicated visual scripts (Chang et al., 2018), are central to our understanding of how learners learn to read a logographic script (Tan et al., 2005a,b; McBride, 2016; Yin and Zhang, 2021). Given wide differences between alphabetic and logographic scripts, learning to read and write Chinese characters is one of the main challenges and a real problem for CSL beginners who are non-native Chinese speakers (Allen, 2008; Ye, 2013; Li, 2020).

In literacy education, the increasing use of electronic media and devices gives rise to a spirited debate centering on the role of Chinese characters: does Chinese reading depend on character writing^1^ (Tan et al., 2005b; Bi et al., 2009)? Is it necessary to learn Chinese character writing (Allen, 2008; Lam and McBride, 2018; Zhou et al., 2020)? When is the best time to start learning Chinese characters? (Ye, 2013; Knell and West, 2017). Over the past two decades, many cross-disciplinary scholars have attempted to solve these problems. These studies mainly focused on character recognition (e.g., Siok and Fletcher, 2001; Liao et al., 2008; Guan and Fraundorf, 2020; Jiang et al., 2020; Zhang H. et al., 2020; Zimmer and Fischer, 2020; Guan et al., 2021), without considering the role of the embodiment of handwriting, which is deemed essential to the embodied cognition theory (ECT). Specifically, the ECT underlines the interaction among perception, action, body, and the environment (Barsalou, 2008). This theory also offers a novel perspective on relationships between language and cognition (that is, the physical body and embodied experience are the origins of cognition), setting it apart from the modular view of linguistic nativism, linguistic determinism, and linguistic relativity, which treats language and cognition as distinct and independent systems.

Some recent studies have established and adopted a large-scale character handwriting database to examine relationships between Chinese character writing and reading. Of these, Wang et al. (2020) collected 200 handwriting characters from 203 native Chinese speakers with their writing latencies, durations, and accuracies. They used this database to explore influential factors of Chinese character amnesia (Huang et al., 2021b) and then further investigated the role of lexical characteristics and individual differences in relationships among tip of the pen (TOP), character amnesia, and partial orthographic access in TOP states (Huang et al., 2021a). However, researchers have used different production protocols and measurements of Chinese character writing and reading. For example, Wong (2018) measured writing fluency using a character copying task with restricted time. In the research of Li et al. (2019), writing was assessed with a dictation task.

This study aimed to examine the role of Chinese handwriting in reading acquisition through big data from a national CSL proficiency test, Hanyu Shuiping Kaoshi (HSK). The primary objective was to investigate how three embodiment factors of a Chinese character, Chinese character writing (bodily activity), language distance (past language usage), and cultural background (bodily coupling with the environment), may contribute to individual differences in CSL reading proficiency. By highlighting the role of character writing in CSL reading acquisition, we hope to contribute to the existing literature for a better understanding of the multifaceted and embodied features of CSL reading acquisition and provide support to the theoretical perspectives of ECT.

### Overview of Chinese Character Research

#### Orthographic Knowledge of Chinese Characters

Researchers generally have acknowledged that sufficient orthographic knowledge benefits the processing of word spelling and reading comprehension (Perfetti and Hart, 2002; Ehri, 2005, 2014; Koda, 2007). However, as a morpheme-syllabic writing system, the orthography of Chinese is noticeably different from grapheme-phoneme writing systems such as English. This difference becomes one of the main challenges in learning Chinese (Shen, 2010; Hsiao et al., 2015; Zhang and Reilly, 2015; Li, 2020). There are two main difficulties that may be caused by this L1–L2 orthographic variance in CSL acquisition.

The first difficulty is due to complicated components of Chinese orthography, which requires lots of cognitive effort to decode, memorize, and distinguish (Qian et al., 2018). Chinese characters can be roughly classified into two categories: compound and integral (Wang et al., 2003; Shen and Ke, 2007; Du, 2015). In general, an integral character has only one radical on its own, and a compound character contains at least two or more radicals. The orthography of almost all Chinese characters consists of three elements: stroke, radical, and whole character (Shen and Ke, 2007). Usually, several strokes constitute a radical and several radicals form a character. Statistically, in modern Chinese, 214 radicals with 32 strokes that convey both sematic and pronounceable information appear in about 80% of 7,000 frequent Chinese characters (McNaughton and Ying, 1999; Chinese Language Committee, 2009).

The second difficulty is the weak correspondence between form and pronunciation of Chinese characters (Shen, 2005; Sung and Wu, 2011). Especially, majority of Chinese characters are homophones. For example, the phonogram “
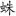
” (pronounced “zhu1,” spider), which consists of the semantic radical “
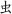
” meaning “insect,” and the phonetic radical “
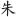
” (pronounced “zhu1”) (the numbers are for indicating tones). Likewise, “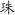,” “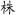,” “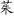,” and “
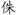
” are all pronounced “zhu1” based on their phonetic radical “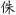.” However, the connection among pronunciation, meaning, and character form is not always regular. In other words, it is diverse for the correspondence between the pronunciation of a phonogram and its combination of radicals. For instance, “
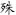
” (pronounced “shu1,” different) is not dependent on its phonetic radical “
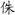
” (zhu1) or its semantic radical “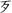,” meaning “evil.” An indexical hypothesis (Glenberg and Robertson, 1999) claims that the most important language comprehension process is indexing of words or scripts to corresponding objects or mental representations. Together, these examples and studies indicate that understanding the underlying schema of Chinese characters could contribute to further Chinese reading comprehension of learners.

In summary, Chinese seems more linguistically complex than other languages might be on account of the unique combination rules of character orthography. However, the complicated features are almost certainly results of the historical evolution of the Chinese language for over thousands of years. As a basic unit of the Chinese language, character, which heavily carries culture, is a semiotic tool to communicate and a medium of cognition and thought.

#### Embodiment of Chinese Characters

Since the 1980s, the idea of embodiment has received increasing emphasis in cognitive sciences. According to the ECT, the medium through which one knows the world is the “body” (e.g., Clark, 1997; Lakoff and Johnson, 1999; Glenberg and Robertson, 2000; Streeck et al., 2011; Glenberg and Gallese, 2012; Wilson and Golonka, 2013). This “body” not only refers to one’s physical organisms but also bodily contact with the world (Inui, 2006). Namely, cognition is assumed to be grounded in embodied experience (embracing bodily activity and perceptual experience) and context. So far, scholars have a different understanding of embodiment. Based on previous literature, the implications of the embodiment of Chinese characters can be discussed in three aspects.

First, handwriting is an embodied activity. The ECT claims that cognitive processing is associated with the particularity of one’s physiological body (Shapiro, 2007; Streeck et al., 2011; Glenberg and Gallese, 2012). In other words, bodily activity might be a way of knowing. It has previously been observed that the perceptuo-motor process of bodily activity offers information and potentially influences the construction of knowledge (Streeck et al., 2011; Lawrence, 2012). For example, study of Strati (2007) described how a building worker skillfully walked on a roof using the tactile experience of his feet.

Thus, a handwritten script is “an imprint of action” (Longcamp et al., 2006). Writing by hand is a direct way by which bodily movements can interact with cognitive processing. In addition, handwriting mobilizes perceptuo-motor processing (Addy, 1996), and then the perception during writing contributes to cognitive processing (Longcamp et al., 2003; Tan et al., 2005b; James, 2017). Interestingly, brain neuroimaging research has found that some specific Brodmann’s areas were strongly activated for Chinese characters (Tan et al., 2001, 2005a; Hu et al., 2018). The findings from these studies suggest that the processing Chinese characters may be related to the experience of bodily activity. If so, handwriting, as an embodied action, might contribute to the cognitive processing of characters.

Second, learning a new language is learning a new conceptual system. Traditional dualism claimed that a physical substance (mind) had no bearing on a mental substance (body). Unlike the traditional view, the ECT supports the view that the experienced sensations and perceptions in the past are still work on one’s cognitive processing in the moment (Lakoff and Johnson, 1980). In short, an embodied experience might shape one’s way of thinking. Couched in the ECT framework, Lakoff and Johnson (1980) proposed that the way one conceptualizes the world is metaphorical. Likewise, there is a strong possibility that language is metaphorical. Therefore, to learn a new language^2^ is likely to learn a new system of metaphors and conceptions apart from language features. Research has also shown that comprehension cannot be devoiced from the bodily sensory-motor system (Desai et al., 2010; Emmorey et al., 2014; Slepian and Ambady, 2014). For example, through tasks on building bodily metaphor, Slepian and Ambady (2014) found interactions among sensorimotor schema, comprehension and estimation, and abstract conception in reading.

Chinese, to some extent, is a representation of the ECT, with enormous metaphors involving embodied experience. Although simplification results in weaker link among radicals, the Chinese written language still represents the linguistic, psychological, and cultural features of the Chinese nation (Pae, 2020). According to Quinn (1987), a cultural model was critical for social members’ understanding of the world and their behavior. Cultural models underlying the conceptualization of Chinese characters reflect culture-specific concepts constructed in Chinese context. So far, researchers have proven the effect of past language usage of bilinguals on cognitive categorization in several conceptual domains, such as speech, gestures, memory for motion, and posture (Brown and Gullberg, 2008, 2011; Filipović, 2011; Smithson and Nicoladis, 2013), objects and substances (Cook et al., 2006; Pavlenko and Malt, 2011), emotions (Pavlenko and Driagina, 2007), motion events (Bylund et al., 2013; Park, 2020), and event construals (Wang and Wei, 2019). Moreover, behavioral and event-related potential (ERP) research has found that various language speakers have different ways of perceiving and conceptualizing the world (Liu et al., 2010; Jared et al., 2013; Wang et al., 2018). For example, Liu et al. (2010) investigated English and Mandarin speakers through category judgment tasks. English speakers showed larger N300 and N400 ERP component differences. In contrast, Chinese speakers showed no such differences in processing atypical and typical items (e.g., to judge the membership of “train” in English or huo3che1 “
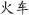
” in Chinese [atypical] and “car” in English or jiao4che1 “
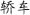
” in Chinese [typical] pictorial exemplars of a category “vehicle” in English or che1 “
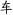
”in Chinese). The researchers further found that the Chinese speakers elicited moderate N300 and N400 effects on Chinese orthographically transparent items (e.g., the radical “bug” chong2 “
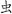
” in character for the noun “butterfly” hu2die2 “
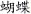
”), while the English speakers showed such ERP effects on English morphologically transparent items (e.g., catfish). These findings indicate dramatic differences in the English and Chinese speakers’ processing of category judgments.

Therefore, one of the essential preconditions for understanding a script or a word is to underlie its metaphors and conceptions. If not, it may cause misunderstanding. Carrying over “
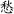
” exemplifies the metaphor of Chinese characters. The metaphor “
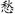
” originated from a Chinese schema that autumn (“
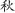
”) is in one’s heart (“
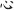
”). In detail, “
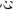
” traditionally represents the physical heart and is the core of mind, thought, and mood [see Yu (2009) for more detailed explanations]. In Chinese culture, autumn is often a metaphor for mixed emotions of happiness of the harvest and sadness of the forthcoming desolate scene. Thus, “
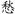
” generally means a complicated feeling of happiness, worry, solitude, and sadness. Compared to the integral characters stated before, “
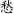
” is beyond simply describing the schema of concrete objects. All of these language idioms or conventions represent the perception and conceptualization of Chinese people in daily life.

Third, cultural background influences cognition of Chinese characters. The ECT believes that interactive relationships exist among our mind, action, body, and context (Lakoff and Johnson, 1980, 1999; Hutchins, 1995; Barsalou, 2008; Spackman and Yanchar, 2014).

In the first place, the development of Chinese characters is embedded in culture and history, and, in turn, characters that carry cultural and linguistic information influence surrounding regions. Historically, Chinese characters were cornerstones of East Asian culture development. Characters documented Chinese culture and then spread throughout other regions for millennia. These regions are considered as a cultural sphere, Sinosphere (also known as the East Asian cultural sphere, Chinese cultural sphere, or Sinic/Sinitic world). On the one hand, there are notable linguistic similarities among Sinosphere languages (Matisoff, 1973, 2001; Enfield, 2011; Brunelle and Kirby, 2016). On the other hand, Sinosphere regions, such as Japan, Korea, and Vietnam, share similar conceptions, which can reflect in Chinese characters.

In the second place, Chinese characters acquisition is affected by bilingual learners’ L1 context. Contextual factors affect and constitute cognition. ERP research has found that bilinguals’ cultural context impacts their semantic (Jared et al., 2013) and conceptual representations (Berkes et al., 2018) in lexical access. To elaborate, we continue with the example “
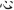
” (heart). Matisoff (1986) discussed the concept of heart and mind in Southeast Asian languages with a comparative perspective in English. The research found that many Sinosphere languages prefer to describe psychological phenomena using metaphors of a bodily organ. For Chinese characters, radical usage, which represents bodily organs, like “
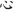
” (heart), gives morpheme a psychological allusion. As mentioned above, the Chinese “heart” covers the meaning of both heart and mind, regarded as the center of both emotion and mood. However, like other European languages, English concepts of the heart are separated from the mind. This difference reflects a culturally specific concept developed in the Anglo-Saxon context (Wierzbicka, 1989, 1992; quoted in Yu, 2009). Therefore, individuals, especially bilinguals (Jared et al., 2013; Berkes et al., 2018; Wang and Wei, 2019), with different L1 sociocultural backgrounds are likely to have different cognitions for characters.

### Research on Chinese Character Writing and Reading Comprehension^3^

#### The Role of Chinese Characters in Reading Comprehension

The role of Chinese characters in reading has received increased attention across a number of disciplines in recent years. Unlike an alphabetic language, the orthographic form of Chinese characters is more effective than phonological representations in reading comprehension because of weak regular corresponding rules between the form and phonology of characters (Xu et al., 2013). Some research has investigated the role of characters by stroke (e.g., Zhang, 2014; Chang et al., 2015; Dall et al., 2021), pinyin (e.g., Zhang et al., 2019; Xiao et al., 2020; Ju et al., 2021), character copy skills (e.g., Ye et al., 2021), phonological awareness (e.g., Siok and Fletcher, 2001; McBride-Chang et al., 2004; Zhang and Roberts, 2019, 2021a), morphological awareness (Nagy et al., 2002; Liu and McBride-Chang, 2010), radical awareness (Shen and Ke, 2007; Wong, 2017; Zhang and Roberts, 2019), and orthographic awareness (Siok and Fletcher, 2001; Loh et al., 2018; Tong et al., 2019; Chen et al., 2021; Zhang and Roberts, 2021a). However, differences in testing methods may lead to different results. For instance, some research (e.g., Tan et al., 2005b; Wong, 2017) employed a copy task in which materials were self-designed to the character writing ability of test participants, and the results indicated close relationships between Chinese handwriting and reading. However, other research (e.g., Zhai and Fischer-Baum, 2019) used a visual same/different judgment task on pairs of characters, and the results failed to find this significant contribution of character writing to reading.

Debates on the role of character writing have gained fresh prominence, with many arguing whether handwriting contributes to Chinese reading. There is a vast amount of research showing that strong knowledge of and skills in character writing facilitate orthographic processing in Chinese reading for children (Tan et al., 2005b; Wang et al., 2014, 2015; Chung et al., 2018; Zhang J. et al., 2020) and adults (Kuo et al., 2015; Tong and Yip, 2015; Tong et al., 2016; Zhang J. et al., 2020). However, literature has emerged that offers contradictory findings on strong effects of character writing on reading (e.g., Bi et al., 2009; Zhai and Fischer-Baum, 2019). For instance, a case study on a brain-impaired adult patient with writing deficit found no such effect of writing through reading and character writing tasks (Bi et al., 2009). Although he lost the ability to write by hand, this patient could still recognize Chinese and read it aloud. This finding displayed a clear dissociation rather than a strong relationship between Chinese writing and reading. However, conflicting results from these studies suggest the need to further investigate if CSL learners with different Chinese character writing skills also have variances in reading acquisition or, more likely, if are there factors that moderate the reading differences and, if so, what are the factors?

The importance of handwriting is now well established. While some research has been carried out on character recognition, there is very little scientific understanding of character writing. For instance, studies have found that visual-orthographic knowledge can help learners more quickly and easily recognize characters (e.g., Tong and McBride-Chang, 2010; Huang et al., 2020). Nevertheless, neuroimaging studies have found that reading Chinese characters activates motor-related zones apart from visual zones in the brain (Longcamp et al., 2003, 2005; Tan et al., 2005b; Yin and Zhang, 2021). In addition, research has also found a moderate effect of writing motor execution on deep orthographic processing (Yin et al., 2020). However, the influence of character writing on reading comprehension in CSL reading acquisition has remained unclear.

#### The Role of Language Learning Experience in Reading Comprehension

Embodied cognition theory research opens an avenue for a better understanding of cognitive processing and the three elements (bodily activity, language learning experiences, and context). Reading comprehension is one of the advanced cognitive processes closely associated with linguistic knowledge and cognitive skills (Stanovich, 2000; [Bibr B78][Bibr B78],^2007^; Jeon and Yamashita, 2014). Recent L1 and L2 reading research has extensively applied the component-skills approach to reading. One much-debated question addressed by Alderson (1984) in reading research was whether L2 language knowledge (e.g., orthographic, vocabulary, and phonological knowledge) was related to reading proficiency or cognitive processing (e.g., attention, working memory and metacognitive awareness). The existing body of research on language experience suggests that prior literacy experience influences literacy development in SLA (Hamada and Koda, 2008; Koda, 2008; Tong et al., 2016), and that language learning experience benefits in the early formation of perceptual organization (Kimchi and Hadad, 2002; Yeh et al., 2003). For example, Yeh et al. (2003) investigated the effect of language learning experience on the perceived graphemic similarity of Chinese characters with two shape-sorting tasks. The results showed that Chinese and Japanese undergraduates categorized characters based on configurational structures. In contrast, Chinese illiterate adults and kindergarteners classified characters by strokes or components. However, what is not yet understood is the relationship between language experience and CSL reading comprehension.

Language distance refers to the extent of similarity or differences between two languages. In other words, language distance is a measurement of classifying languages by their linguistic features. The current understanding of language distance in SLA suggests a positive cross-linguistic influence when L1 and L2 are linguistically similar or overlapped (Sharwood Smith and Kellerman, 1986; Jarvis and Pavlenko, 2008), and negative influence more likely occurs in beginners (Odlin, 1989). This influence may happen at a conceptual level (Jarvis and Pavlenko, 2008). According to Koda’s 2005a,b Transfer Facilitation Model, language transferring ability can provide top-down help for reading development and other associated abilities in another language. Because of overlaps and differences among all languages, language distance might be a main factor of the transfer.

Evidence from several studies suggest that language distance plays a moderate role in L2 reading acquisition (Melby-Lervåg and Lervåg, 2011; Jeon and Yamashita, 2014). A meta-analysis research (Jeon and Yamashita, 2014) study showed that linguistically similar languages (between Indo-European L1s and L2s) were more closely related to reading comprehension than linguistically distant languages. Interestingly, in the study of Jeon and Yamashita, language distance significantly affected reading comprehension but had no moderating effects on vocabulary or grammar knowledge, thereby rejecting their hypothesis. The authors explained that observing the cross-linguistic influence might be easier at a complex variable level (e.g., reading comprehension) than at a single variable level (e.g., vocabulary and grammar). Meanwhile, Tong et al. (2016) proposed a non-native Chinese character processing (NCCP) model to explain how semantic and phonetic radical information was accessed when learners with different L1 orthographies were processing Chinese characters. In this model, L1–L2 (Chinese) orthographic distance and context modulated the activation of word identification in two language systems. However, there has been little agreement on how L1–L2 distances of learners moderate the relationship between Chinese character writing and reading comprehension.

#### Role of Context in Reading Comprehension

Sociocultural context is pivotal in SLA research (Vygotsky, 1978; Ellis, 2008; Green and Abutalebi, 2013; Tong et al., 2016). There are some interfaces between sociocultural theory (Vygotsky, 1978) in SLA and ECT in cognition: they both focus on the interaction between individuals and environments (Lan et al., 2015). According to the theory of Vygotsky, relationships between individuals and social context are inseparable; context and interaction with the context are two critical mediating factors in language learning (Ellis, 2008). Interacting in the social context provides essential SLA scaffolding (Vygotsky, 1978). This theory emphasizes the importance of external mediation, from which internal activity originates (Swain, 2000; Ellis, 2008). However, only few studies have investigated the role of character writing in Chinese reading acquisition based on these two crucial theoretical frameworks.

Theories on L2 reading have focused on predictors of social environment. For example, the Component Model of Reading (Joshi and Aaron, 2000, 2012) integrated individual and contextual factors. This model proposed that ecological and psychological surroundings had an impact on reading proficiency and cognitive skills. Moreover, recent bilingualism research has suggested ascribing language processing, cognitive processing, and brain organization to the experience of language learning and using and social factors surrounding this experience (Gullifer et al., 2018, 2021; Anderson et al., 2020; DeLuca et al., 2020; Tiv et al., 2020).

When it comes to Chinese characters, it is hard to overstate the importance of Sinosphere, because Sinosphere reflects the continuous improvement of East and Southeast Asian civilization around Chinese language and culture. Many previous studies have pioneered the effect of language background and compared CSL characters learners in various language contexts (Ke, 1998; Jiang, 2003; Xu, 2007; Zhang, 2008; Lin and Collins, 2012; Li et al., 2014; Ke and Chan, 2017; Tang and Chan, 2021; Zhang and Roberts, 2021b). Of these studies, Zhang (2016) compared the development of character orthographic awareness between ethnic and non-ethnic Chinese international students from Southeast Asian countries. The results failed to showed advantages of ethnic Chinese in the development of CSL character orthographic awareness but of character component and position awareness. Ke and Chan (2017) explored relationships among CSL reading strategies, L1 background, and L2 proficiency by examining participants from Chinese and non-Chinese cultural spheres. The results showed that intermediate learners with Chinese cultural background were more advantaged than the others, but this advantage disappeared when learners were more proficient in Chinese. These findings suggest a difference in L2 achievement in different L1 cultures.

### This Study

Based on literature review, enormous research has documented the role of Chinese characters in CSL reading acquisition. However, researchers have paid little attention to the relationships between characters and reading based on the perspective of ETC and SLA from big data in a real scene. First, writing is an embodied activity, but most research on the role of Chinese characters in reading has investigated character recognition instead of character writing, and studies on character writing have adopted unofficial measurement tools or tasks to test the character writing skills of participants. Second, Chinese characters are embodied in culture and context. Although ECT frameworks have been discussed theoretically in most previous research studies, empirical evidence is still lacking. Third, since there are overlaps between the ECT and SLA, it is indispensable to employ the ECT to examine SLA issues, despite lack of previous studies. Therefore, to fill these gaps, this study focused on the following questions:

RQ1: Does character writing (i.e., bodily activity) facilitate reading?

H_1_: Character writing proficiency is positively related to the reading proficiency of learners.

RQ2: How do the L1s of learners (i.e., past language experience) moderate relationships between character writing and reading?

H_2_: Character writing proficiency will be more strongly associated with decrease in reading proficiency of learners when their L1s are linguistically distant to Chinese than when their L1s are more similar to Chinese.

RQ3: What role does cultural background (i.e., environment) play in the CSL reading comprehension of learners with various cultural backgrounds?

H_3_: There will be an interactive effect between cultural background and handwriting. Character writing proficiency will be more strongly associated with an increase of reading proficiency of Sinosphere learners than non-Sinosphere learners.

## Materials and Methods

### Participants

We made use of the subset of a large-scale database, which was gathered in 2009, containing information on 80,506 examinees who participated in the HSK test at various locations in China. A brief questionnaire collected their individual background information when examinees registered for the HSK test online. This non-mandatory survey included questions about basic demographic features of learners such as gender, country of birth, age, and mother tongue.

Next, we selected samples from the original data of 80,506 examinees. First, 4,018 participants were excluded because of missing individual information and 1,728 participants owing to their unrealistic or ambiguous answers in the questionnaire. Second, 17 participants were excluded because of the scribble of the names of their L1s. Third, the study also removed 381 participants whose age was over 80 years. The final samples consisted of 74,362 CSL learners (*M* age = 23.3 years, range = 9.4–79.3 years; 36,528 males and 37,834 females) without outliers (e.g., unrealistic or ambiguous answers) or missing values.

The learners spoke 67 L1s (*M* = 1,109.9 speakers per language) and came from 173 countries (*M* = 429.8 speakers per country). There were 307 learners with Chinese as an L1, accounting for 0.41% of all the samples. These L1s, according to the World Atlas of Language Structures [WALS] (Dryer and Haspelmath, 2013), included about 14 language families (i.e., Afro-Asiatic, Altaic, Austro-Asiatic, Austronesian, Dravidian, Indo-European, Japanese, Kartvelian, Korean, Niger-Congo, Sino-Tibetan, Tai-Kadai, Turkic, and Uralic).

### Instruments

#### Hanyu Shuiping Kaoshi

Hanyu Shuiping Kaoshi (HSK, abbreviation of Chinese pinyin: Hànyǔ Shuǐpīng Kǎoshì; literally translated as Chinese Proficiency Test) is a standardized and the most widespread test for assessing the Chinese language proficiency of non-native speakers in the world. HSK development has gone through three stages (see review by Teng, 2017 and Meyer, 2014). The HSK Testing Center of Beijing Language Institute (BLCU) designed and developed this test in 1984. Then, in 2010, China’s Hanban/Confucius Institute Headquarters (i.e., Office of Chinese Language Council International) supported and reformatted the test as the New HSK. The designers kept all the three versions of HSK comparable (Hanban, 2011).

In this study, CSL reading and writing data came from the raw data of HSK (Elementary–Intermediate) (*M* = 50, *SD* = 15) in 2009.^4^ Why did we use the sample data of BLCU’s HSK rather than the New HSK? First, the BLCU’s HSK has a longer history. The BLCU’s HSK had advanced based on experience in CSL proficiency testing design and practice of over 26 years (1984–2010). During this time, researchers conducted many empirical investigations to improve the test (e.g., Chai, 2006; Cheng, 2006; Huang, 2006). Thus, these studies verified the BLCU’s HSK reliability and validity (Meyer, 2014). Second, the BLCU’s HSK mainly focuses on examinees’ integrative ability of four language skills (listening, speaking, reading, and writing) and communicative competence, which is more applicable for this study.

The HSK consists of three levels of CSL proficiency: beginning level (HSK Basic), intermediate level (HSK Elementary–Intermediate), and advanced level (HSK Advanced). Learners who have mastery of 2,000–5,000 Chinese words and certain grammar rules are at the HSK Elementary–Intermediate level. This level covers learners with a wider range of proficiency than other HSK levels. HSK Elementary–Intermediate consists of four subtests (i.e., listening [50 items], grammar structure [30 items], reading [50 items], and cloze [40 items]), with 170 items in total. Of these, 1–154 items are multiple choice and 155–170 items are fill-in-the-blanks. This study focused on the results of two sections: the reading section and character writing part of the cloze section.

### Variables

In total, this study inspected six variables: one dependent variable (CSL reading proficiency), two control variables (gender and age), and three independent variables (an individual-level variable, Chinese character writing proficiency [CCWP], and two group-level variables, language distance and cultural background).

#### Chinese as a Second Language Reading Proficiency

**FIGURE 1:**
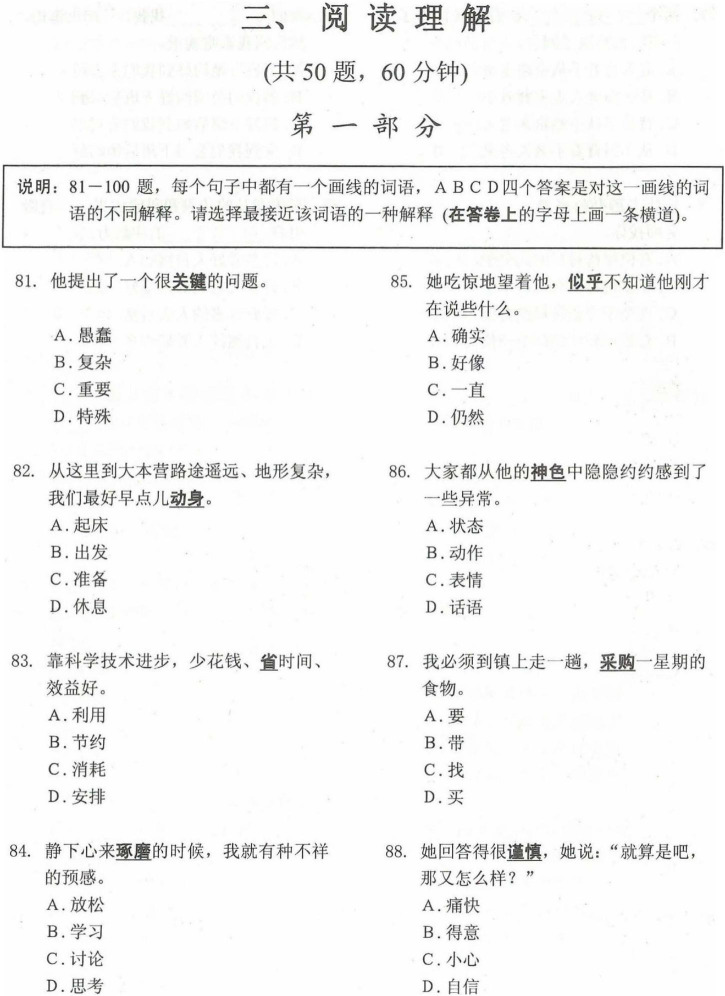
Sample test in the first part of the Hanyu Shuiping Kaoshi (HSK) reading test.

**FIGURE 2:**
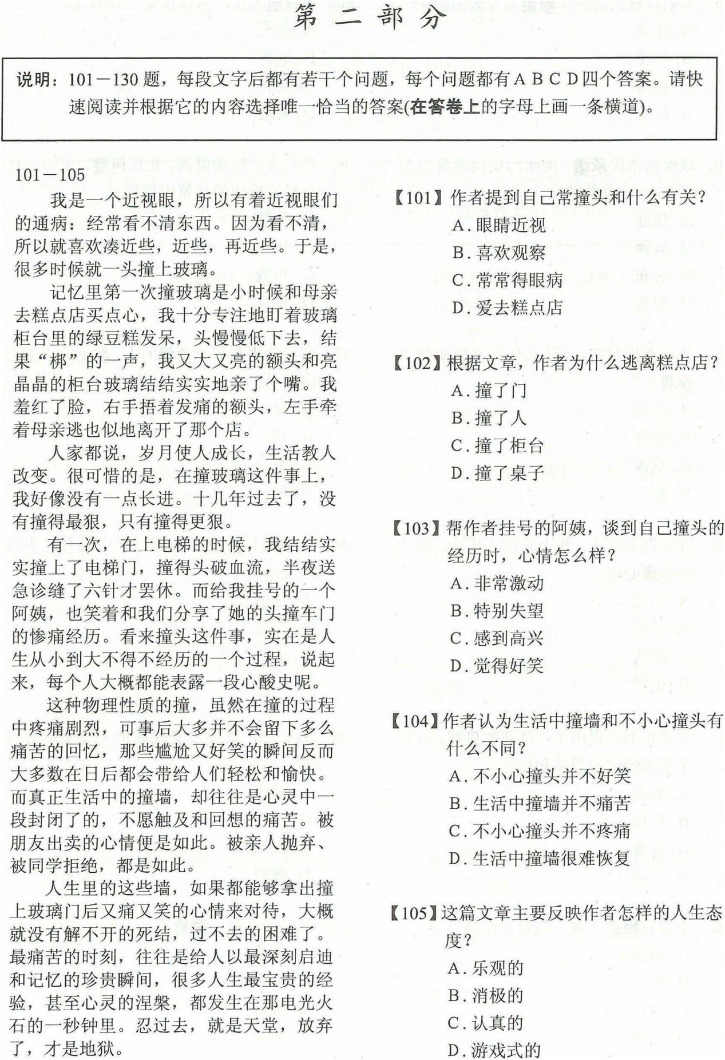
Sample test in the second part of the HSK reading test.

The data on CSL reading proficiency came from the results of the HSK reading test. The whole HSK reading section lasted 60 min and consisted of two parts: word substitution and paragraph reading. The first part examined the ability of students to understand, recognize, and use words in specific contexts. Within the first part, the test gave 20 sentences with words underlined. Then, examinees chose the best words with the most similar meaning and usage from four options to replace the underlined word in each sentence. The second part examined the competence of students in paragraph reading comprehension and reading speed within a given time. For example, competence consisted of summarizing and interpreting texts, extracting keywords and sentences, deducing implicit information, and identifying attitudes, moods, opinions, or intentions of authors. The test contained six to eight short articles of varying lengths, difficulties, forms, and topics. In addition, examinees answered 30 multiple-choice questions (one point per item), Figures 1, 2, respectively, show sample tests in the first and second parts of the HSK reading test.

#### Chinese Character Writing Proficiency

**FIGURE 3:**
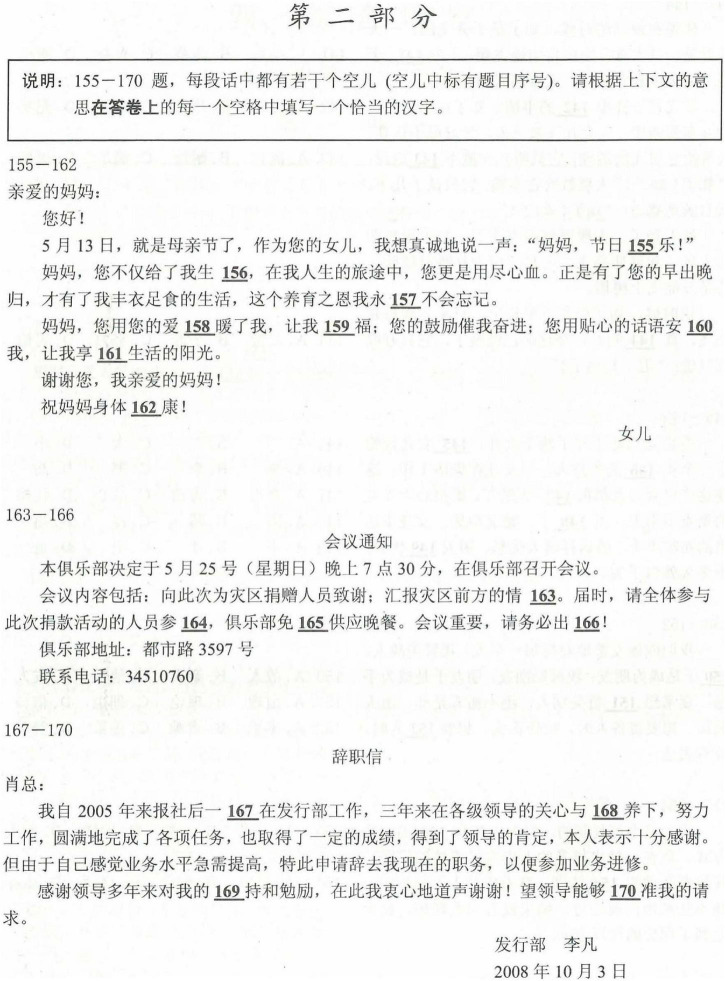
Sample test in the HSK character writing test.

The study used the phrase “CCWP” to refer to Chinese character writing test scores that students got in the HSK test. The second part of the HSK cloze section was the basis of the CCWP data. This part mainly examined students’ CSL orthographic competence of mastery and usage of lexical words and writing. Test-takers filled in 16 blanks (one point per blank) within the orthography part with appropriate and correct handwritten Chinese characters based on three to four passages that were give. The whole cloze section lasted for a total of 30 min. Figure 3 shows a sample test in the HSK character writing test. The CCWP was divided into two groups with low and high writing proficiency (baseline = low level, score lower than an HSK character handwriting test score of 7/16).^5^

#### Language Distance

This study computed the language distance between participants’ L1s and Chinese through the Automated Similarity Judgement Program (ASJP) database. ASJP is an extensive cross-linguistic linked database belonging to the Max Planck Institute. The latest version (19th edition) of ASJP (Wichmann et al., 2020) covers 40-item word lists of 5,499 languages. These lists cover nearly 70% of the world’s extant languages.

With consideration for language diversity, this study adopted the L1s of participants rather than the official languages of their nationalities. By applying the ASJP program (Bakker et al., 2009), we calculated language distance values using the Levenshtein Distance method comparing phonetic forms of 40 core words (Holman et al., 2008; see Table 1). The less linguistically similar an L1 is to Chinese, the higher the ASJP value. The language distance ranged from 0 to 102.88 (*M* = 97.92, *SD* = 6.7).

**TABLE 1:** Descriptive statistics of participants with language distance between L1s and Chinese.

Family	L1	*N*	Linguistic distance	Family	L1	N	Linguistic distance
Afro-Asiatic	Amharic	8	100.99	Indo-European	German	738	102.35
	Arabic	767	101.49		Greek	19	102.88
	Hebrew	36	102.77		Hindi	853	102.83
	Somali	37	99.81		Italian	611	100.33
Altaic	Azerbaijani	39	100.60		Latvian	9	94.76
	Kazakh	1,886	99.61		Lithuanian	11	99.03
	Mongol/Khamnigan	2,931	99.48		Marathi	5	101.81
	Turkish	634	101.63		Nepali	206	100.20
	Turkmen	137	101.61		Norwegian	28	102.12
	Uyghur	430	99.35		Persian	47	100.93
	Uzbek	98	99.96		Polish	121	102.23
Austro-Asiatic	Khmer/Cambodian	149	100.52		Portuguese	187	101.75
	Vietnamese	4,369	101.31		Romanian	37	101.05
Austronesian	Fijian	4	98.95		Russian	4,550	100.28
	Indonesian	1,991	100.62		Serbian	16	101.15
	Malagasy	6	100.26		Sinhala	74	99.49
	Malay	55	101.41		Slovak	23	102.64
	Samoan	27	100.34		Spanish	632	98.66
	Tagalog/Filipino	45	94.68		Swedish	101	100.88
Dravidian	Tamil	38	99.49		Tajik	114	100.58
	Telugu	497	100.28		Ukrainian	146	97.08
Indo-European	Albanian	15	100.97		Urdu	592	100.17
	Armenian	19	98.11	Japanese	Japanese	11,124	98.40
	Assamese	5	100.16	Kartvelian	Georgian	11	99.43
	Belarusian	9	101.81	Korean	Korean	30,408	95.91
	Bengali	50	101.62	Niger-Congo	Swahili	52	99.93
	Bulgarian	66	99.81	Sino-Tibetan	Burmese	160	100.79
	Catalan	4	100.50		Chinese	307	0.00
	Czech	60	102.01	Tai-Kadai	Lao	710	100.8
	Danish	37	101.48		Thai	3,696	99.06
	Dutch	104	100.14	Turkic	Kyrgyz	171	99.53
	English	2,518	102.40	Uralic	Finnish	50	98.58
	French	1,446	101.86		Hungarian	36	101.43

*Sort in alphabetical order.*

#### Cultural Background

Based on previous studies (Pae, 2020; Tang and Chan, 2021), Sinosphere contains five entities: China, Japan, North Korea, South Korea, and Vietnam. Some researchers prefer to divide Sinosphere into two categories: the “Chinese cultural sphere” and the “non-cultural sphere.” Others include South Asian countries (such as Laos and Singapore), with the limited L1 of learners influenced by the Chinese language and culture. For instance, they do not use Chinese characters in their language systems (B. Zhang, 2020). However, the dichotomy overlooks the individual differences caused by the influence of the L1 backgrounds of learners, CSL environments, and cultural factors (Li and Zhang, 2021). Thus, this study divided the sample by participants’ language context of current residence into three groups: the narrow-Sinosphere (NSG, i.e., learners from Japan, North Korea, South Korea, and Vietnam), broad-Sinosphere (BSG, i.e., learners from 10 Southeast Asian countries: Laos, Cambodia, Thailand, Burma, Malaysia, Singapore, Indonesia, Brunei, the Philippines, and East Timor) and non-Sinosphere (non-SG, i.e., learners from all other countries) groups (baseline = NSG).

#### Control Variables

Previous research has suggested that differences between males and females and children and adults may influence L2 reading and writing development (Phakiti, 2003; Jeon and Yamashita, 2014; Van der Slik et al., 2015; Vilas et al., 2019). However, reasons for the variation are still under investigation. This study controlled for the gender and age demographics of the examinees. Following previous research, two controlled variables were encoded as dichotomous, specifically, males as 1, females as 0, children (under 18 years old) as 0, and adults as 1.

### Data Analysis

This study employed a two-level hierarchical linear model (HLM) to investigate the contributions and interaction effects of individual- and group-level variables on CSL reading (Hox, 2010). HLM is a statistical technique to analyze variance in the dependent variables by modeling the hierarchy of the nested structure of the data; for example, in this study, learners in a country shared variance of reading proficiency according to their common L1s. Considering within- and between-group regressions, this study used HLM software version 6 (Raudenbush et al., 2006) with a restricted maximum likelihood method to depict how the individual- and group-level predictors affected the reading proficiency of the students. Both the categorical variables (CCW and cultural background) were dummy-coded and added in the equation uncentered. All continuous variables involved in interaction effects were grand mean centered to avoid multicollinearity (e.g., Hofmann and Gavin, 1998; Enders and Tofighi, 2007).

Practically, the process of hierarchical model establishment could be divided into four steps (Heck et al., 2013): (1) null model, (2) individual-level (or level 1) random coefficient regression model, (3) group-level (or level 2) means-as-outcomes regression model, and (4) full model. Null model was regarded as the baseline model without any predictors for subsequent model comparison. The null model developed the levels 1 and 2 models by adding variables at each hierarchical level. The final model included full variables of the two levels for investigating statistical relationships with reading proficiency.

The level 1 equation is as follows:

Y_i*j*_ = β_0*j*_ + β_1*j*_ (*GENDER*_*ij*_) + β_2*j*_ (*AGE*_*ij*_) + β_3*j*_ (*CCWP*_*ij*_) + *r*_*ij*_,

where *Y*_*ij*_ is the reading test score for student *i* within L1 unit *j*, β_0_*_*j*_* is the level 1 intercept (overall mean scores in the reading test for students with various genders and handwriting levels). β_1*j*_, β_2*j*_, and β_3*j*_ are, respectively, the regression coefficients (slopes) for the two dichotomous control variables GENDER and AGE, and *r*_*ij*_ presents the residual error of the equation.

The level 2 equation of the random intercept model for estimating level 1 means is:

β_0*j*_ = *γ*_00_ + *γ*_01_ (*ASJP*_*j*_) + *γ*_02_ (*BSG*_*j*_) + *γ*_03_ (*non*−*SGj*) + μ_0*j*_,

where γ_00_ is the level 2 intercept (i.e., overall mean scores of reading test for an L1 unit *j*). γ_01_ is the average slope coefficient and predicts the change in β_0_*_*j*_* for one standard deviation change in language distances (ASJP). γ_02_ and γ_03_ are the expected changes of two predictors BSG and non-SG in β_0_*_*j*_* (compared with NSG). μ_0_*_*j*_* is the residual variance of L1 unit *j* after controlling for GENDER. The level 2 model for estimating level 1 slopes with cross-level interaction for two levels is:

β_1*j*_ = *γ*_10_ + μ_1*j*_,

β_2*j*_ = *γ*_20_ + μ_2*j*_,

β_3*j*_ = *γ*_30_ + *γ*_31_ (*ASJP*_*j*_) + *γ*_32_ (*BSG*_*j*_) + *γ*_33_ (*non*−*SG_j_*) + μ_3*j*_,

where γ_10_, γ_20_, and γ_30_ are, respectively, the variances of the two control variables (GENDER and AGE) and level 1 predictor CCWP in the reading proficiency slope across L1s. The term “_*γ*31_(ASJP_*j*_) + _*γ*32_(BSG_*j*_) + _*γ*33_(non-*SG*_*j*_)” states that the relationship between reading proficiency (Y) and handwriting level (X) of an individual depends on language distance (ASJP) and cultural background (BSG and non-SG). μ_1_*_*j*_*, μ_2_*_*j*_*, and μ_3_*_*j*_* represent the residual variance of the L1 unit *j* on the predicted GENDER slope (β_1_*_*j*_*), AGE slope (β_2_*_*j*_*), and CCWP slope (β_3_*_*j*_*).

## Results

### Descriptive Statistics

For the combined sample of 74,362 CSL learners, the overall mean scale scores were 27.76 (*SD* = 10.17) and 6.52 (*SD* = 4.27) for the HSK reading and handwriting tests, respectively. For children (age < 18), mean scores in the reading and writing tests were 25.55 (*SD* = 9.81) and 6.30 (*SD* = 4.06), respectively. For adult learners, the mean scores in the two tests were 28.11 (*SD* = 10.18) and 6.55 (*SD* = 4.30), respectively. At the group level, for the sample of 67 L1s, the mean language distance was 98.79 (*SD* = 12.38).

Before HLM analysis, this study tested the multicollinearity of all the variables. Results of variance inflation factors (VIFs) (all VIFs < 3) show that there was no multicollinearity in the model (Tabachnick and Fidell, 2013). Table 2 summarizes the description of these variables.

**TABLE 2:** Descriptive statistics for the dependent variable (Chinese as a second language, CSL, reading proficiency) and explanatory variables (67 L1s from 173 countries).

			Children (*N* = 8,053)		Adult (*N* = 38,464)	
			Mean	*SD*	Mean	*SD*
NSG (*N* = 46,517)	CSL reading proficiency		26.35	9.42	30.61	9.44
	CCWP		6.67	3.91	7.73	3.95
	Age		16.01	1.69	25.39	7.39
	Language distance		95.76	5.71	97.05	5.02
			*N*	%	*N*	%
	Gender	Male	4,756	59%	18,539	48%
		Female	3,297	41%	19,925	52%
BSG (*N* = 6,897)			Mean	*SD*	Mean	*SD*
	CSL reading proficiency		25.45	10.61	26.31	9.22
	CCWP		6.44	4.35	6.47	4.11
	Age		16.57	1.48	22.90	4.20
	Language distance		98.70	11.20	98.82	9.79
			*N*	%	*N*	%
	Gender	Male	257	46%	2,198	35%
		Female	301	54%	4,141	65%
Non-SG (*N* = 20,948)			Mean	*SD*	Mean	*SD*
	CSL reading proficiency		21.49	10.42	23.74	10.29
	CCWP		4.34	4.14	4.23	4.05
	Age		16.68	1.45	22.91	4.40
	Language distance		98.78	10.93	100.19	7.43
			*N*	%	*N*	%
	Gender	Male	860	54%	9,918	51%
		Female	727	46%	9,443	49%

*1. CCWP is the abbreviation of Chinese character writing proficiency; 2. NSG (Narrow Sinosphere Group) includes Japan, North Korea, South Korea, and Vietnam; BSG (Broad Sinosphere Group) includes Laos, Cambodia, Thailand, Burma, Malaysia, Singapore, Indonesia, Brunei, the Philippines, and East Timor; Non-SG (Non-Sinosphere Group) includes countries outside East Asia.*

### Model Specifications

This study started with a basic model, the null model. The null model involved only intercept items to account for how much of the variance in reading scores lay between the L1 units in the sample. According to the results of the unconditional model, the average reading test score across L1 units was 24.97, *p* < 0.001. The between-language variation, τ_00_ = 21.56, χ^2^ (66) = 19,519.34, *p <* 0.001, indicated significant differences among learners’ L1s in their mean scores in reading proficiency. The within-language variation was σ^2^ = 88.10. The intraclass correlation coefficient (ICC1) estimates the proportion of group-level variance in the population. Thus, when ICC1 ≥0.10, it is valid to use HLM (Raudenbush and Bryk, 2002). In this study, ICC1 = $\frac{\sigma_{{\text{μ}}_{0}}^{2}}{\sigma_{{\text{μ}}_{0}}^{2}+\sigma_{e}^{2}}$ = 21.56/(21.56 + 88.10) = 0.20. Therefore, the results indicated that of the 0.36 (τ_00_+σ^2^ = 0.36) variance in reading scores, the variance caused by group differences was 21.56, accounting for 20% of the overall variance. In comparison, the other 80% of the variance came from individual differences. The estimated inter-rater reliability (ICC2) was 0.90. Such substantial reliability meant a reliable estimation of these models. Thus, adopting multilevel analysis was valid.

Next, level 1 predictors (gender, age, and CCWP) were added into the baseline model as a random coefficient regression model. No group-level predictors were entered this model. Except for age (0.46, *SE* = 0.44, *p* > 0.05), the effects of both gender (−0.56, *SE* = 0.18, *p* < 0.01) and CCWP (12.50, *SE* = 0.36, *p* < 0.001) were highly significant, i.e., female learners may get better reading proficiencies than male learners. Thus, the higher the CCWP of learners, the better their reading proficiency. Children did not differ in reading proficiency from adult learners.

Then, we estimated the means-as-outcomes regression model with only group-level predictors. This model regressed average reading proficiency on language distance and variant cultural backgrounds. The effect of language distance (−0.062, *SE* = 0.01, *p* < 0.001) was significant, indicating that the more linguistically similar learners’ L1s and Chinese, the better their proficiency in CSL reading. Compared with NSG learners, learners from Southeast Asian countries (−5.30, *SE* = 2.98, *p* > 0.05) had no significant variance in reading from NSG learners. However, learners from non-Sinosphere countries (−9.64, *SE* = 2.18, *p* < 0.001) scored lower in reading than the NSG learners.

Finally, we estimated the full model with individual-level and group-level variables (see Table 3 for results of the final model). The level 1 model included the intercept and three slopes: gender, age, and CCWP. These three variables also served as a predictor of level 1 means and language distance, NSG, BSG, and non-SG s slopes in the level 2 model.

**TABLE 3:** Final hierarchical linear models (HLMs) predicting reading proficiency.

Fixed effects	Null Model		Level 1 Model		Level 2 Model		Full Model	
	Coefficient (*S.E.)*	T-ratio	Coefficient (*S.E.)*	T-ratio	Coefficient (*S.E*.)	T-ratio	Coefficient (*S.E*.)	T-ratio
**L1s mean (*β*_0*j*_)**								
Base (*γ*_00_)	24.97 (0.59)	42.06***	21.26 (0.53)	40.36***	39.35 (1.43)	27.58***	24.68 (0.62)	39.60***
ASJP (*γ*_01_)					−0.06 (0.01)	−5.66***	−0.06 (0.01)	−6.39***
Broad Sinosphere (*γ*_02_)					−5.31 (2.98)	−1.78	−6.62 (2.64)	−2.50**
Non-Sinosphere (*γ*_03_)					−9.64 (2.18)	−4.42***	−10.71 (1.92)	−5.56***
**Gender (*β*_1*j*_) (Male = 1)**								
Base (*γ*_10_)			−0.56 (0.18)	−3.11**			−0.48 (0.18)	−2.75**
Age (*β*_2*j*_) (Adult = 1)								
Base (*γ*_20_)			0.46 (0.44)	1.03			0.47 (0.45)	1.04
CCWP (*β*_3*j*_) (high level = 1)								
Base (*γ*_30_)			12.50 (0.36)	35.17***			12.48 (0.34)	36.73***
ASJP (*γ*_31_)							−0.06 (0.00)	−12.03***
Broad Sinosphere (*γ*_32_)							−0.12 (0.81)	−0.15
Non-Sinosphere (*γ*_33_)							0.72 (0.65)	1.12

**Random effects**	**Variance (*SD*)**	**Chi-square**	**Variance (*SD*)**	**Chi-square**	**Variance (*SD*)**	**Chi-square**	**Variance (*SD*)**	**Chi-square**

Between–L1s means (τ_00_)	21.56 (4.64)	19,515.67***	9.32 (3.05)	468.27***	12.01 (3.47)	12,496.10***	16.62 (4.08)	5491.26***
Gender slope (τ_10_)			0.81 (0.90)	177.50***			0.75 (0.87)	182.79***
Age slope (τ_20_)			4.43 (2.15)	1,025.48***			3.60 (1.90)	677.82***
CCWP (τ_30_)			4.63 (2.10)	536.02***			4.40 (2.10)	529.31***
Within–L1s (σ^2^)	88.10	9.39	58.65 (7.66)				58.65 (7.66)	
Deviance	544,198.22		514,166.28		544,252.92		514,178.90	

1. CCWP is the abbreviation of Chinese character writing proficiency; 2. Values of final estimation of fixed effects are reported with robust standard errors; 3. *p < 0.05, **p < 0.01, and ***p < 0.001; 4. NSG is the reference category in the analysis.

### Final Explanatory Models

#### Estimating the Means

The main purpose of the final model analysis was to explore the main and interaction effects of CCWP, language distance, and cultural background on the reading proficiency of the learners after controlling for gender and age. In Table 3, the L1 mean base value (*γ*_00_ = 24.68, *SE* = 0.62, *p* < 0.001) indicates the average reading score for the reference student. This reference student was an adult female with a narrow Sinosphere cultural background and an average language distance between L1 and Chinese and mastered high Chinese character writing skills. The mean reading score of this student was 24.68.

H_1_ addressed the relationship between CCWP and reading proficiency. The regression coefficient for CCWP was 12.48 (*γ*_30_ = 12.48, *SE* = 0.34, *p* < 0.001). Since CCWP was coded as 0 = low and 1 = high, this meant that, on average, learners with high CCWP scored 12.48 points higher on the reading test. Accordingly, this finding supported H_1_.

The language distance was negatively correlated to the average reading score (*γ*_01_ = −0.06, *SE* = 0.01, *p* < 0.001). This result meant that the average reading scores should decrease by 0.06 scale points, each standard deviation higher on the language distance. This prediction does not seem very much, but the language distance in this study ranges from 0 to 102.88, so the predicted difference between the closest and most distant languages is (102.88–0) × 0.06 = 6.17 points on the reading test. This study also observed that, on average, the NSG learners outperformed their BSG and non-SG counterparts by 6.62 and 10.71 points, respectively. For the controlled variables, the results indicated a negative relationship between gender and reading score *γ*_10_ (=−0.48, *SE* = 0.18, *p* < 0.01), but there was no significant difference between children and adult learners (*γ*_20_ = 0.47, *SE* = 0.45, *p* > 0.05).

#### Estimating the Slopes

In this model, we also added interaction effects to test if the influence of CCWP still held. Table 3 also displays the unique effects associated with the interactions of CCWP on reading proficiency.

H_2_ addressed the moderating effects of cultural background. Unexpectedly, this study found no interactions for cultural background. This result indicated that considering the main effects together, the beneficial effects of cultural background factors and CCWP were neither in conflict nor synergistic to learners’ improvement in reading proficiency (the Discussion section addresses this further). Therefore, the findings did not support H_2_.

**FIGURE 4:**
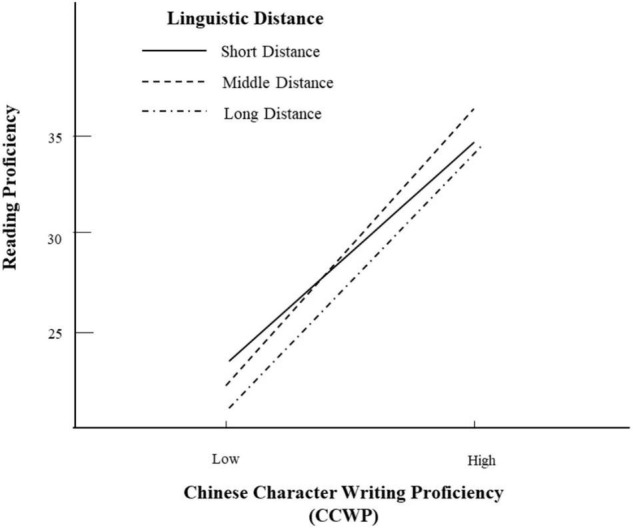
Moderating effect of language distance on reading proficiency.

H_3_ addressed the moderating effects of language distance. The results revealed that there was a negative interaction between language distance and CCWP (*γ*_31_ = −0.06, *p* < 0.001). Figure 4 provides a visual representation of this interaction, signifying that the effect of CCWP on reading proficiency can range from curbing to favorable, depending on the language distance between L1s and Chinese. Specifically, each standard deviation increase in language distance predicted a slight score decrease by 0.06 points, which was small but significant, in reading text for learners with high handwriting skills. These results suggested that learners proficient in handwriting still held their advantage in reading when their L1s were more distant from Chinese. Thus, this finding supported H_3_.

Moreover, outcomes of random effects showed the most variance between learners (σ^2^). This result indicated that individual differences of learners determined reading difficulty more than features of their L1s. This finding was not surprising, since learners succeed in reading development more through embodied activities, especially handwriting, than linguistic features. Interestingly, there was also variance between children and adults, although it was not significant. This finding may be due to the fairness of HSK design. Future research could explore more age-related factors to parse out this variance.

Finally, the model reported an σ^2^ of 88.10 in the full model and 58.65 in the baseline model. According to these results, the effect size of explained variance (Cohen’s *d* = 0.33) for the full model was moderate (cutoff range: 0.15 < *d* ≤ 0.35) (Cohen, 1988). This finding meant that the slope variance reduced sharply after introducing the predictors of two levels and interactions in the null model.

## Discussion

Although the role of the embodiment of Chinese characters in Chinese reading has been widely noted, only few studies involve HSK data on variant language backgrounds. This study was designed to investigate how three factors of Chinese character embodiment, CCWP, language distance, and cultural background, affected Chinese reading proficiency among 74,362 learners from 67 different L1s. First, this study found significant contributions of CCWP, language distance, and cultural background in reading proficiency. Second, there was a significant moderating effect of language distance. After controlling for gender and age, learners who were proficient in handwriting still held their advantage when their L1s were more distant from Chinese. Third, it was unexpected that cultural background had beneficial effects rather than no moderating effects on reading proficiency. These results broadly accord with the ECT.

### Relationships Between Chinese Handwriting and Reading Comprehension

RQ1 set out to explore the positive influence of character writing on reading comprehension. First, as expected, CCWP showed a beneficial effect on the reading proficiency of learners. This finding further support the idea of the ECT and indicate that bodily graphomotor may facilitate the advanced cognition processing in reading (e.g., understanding, memorization, interpretation, and reasoning). This finding also concurs with the view of sociocultural theory (Vygotsky, 1978), which states that internal activity originates from external mediation (Swain, 2000; Ellis, 2008).

Notably, this finding does not mean it rejects the case study of Bi et al. (2009). On the contrary, this study attempted to provide a possible explanation for the debate. A possible explanation for these different results in the debate may be lack of adequate attention to the embodiment of Chinese characters. Thus, the experience of bodily activity and perception could couple with context as metaphors stored in one’s mind (Leung et al., 2011). Due to this, the brain-injured patient could holistically extract the phonetic, morphological, and ideographic information of Chinese character forms after his brain injury. Thus, we rigorously claim that at least for elementary and intermediate CSL learners, character writing may be essential in reading development (Tan et al., 2005b; Lam and McBride, 2018).

Second, this study was unable to demonstrate the interaction effect of age. In line with previous research supporting the writing-to-reading issue, the results of this study suggest a close association between Chinese handwriting and reading in adults and children (e.g., Tan et al., 2005b; Guan et al., 2021; Yin and Zhang, 2021). This result may be explained by the fact that early writing experience could aid the holistic processing of character visual recognition for children (James, 2017) and adults (Tso et al., 2011).

### Role of Language Distance in the Relationships Between Chinese Handwriting and Reading Comprehension

The RQ2 in this study attempted to answer the question of how past language experience (L1 background) moderated the relationship between character writing and reading. Cross-linguistic influence is always an inevitable topic in SLA. As expected, language distance negatively moderated the positive effect between CCWP and reading proficiency, which corroborated L1–L2 transfer theories that similar linguistic features between L1 and L2 promoted SLA achievement (Sharwood Smith and Kellerman, 1986; Odlin, 1989). In addition, these findings also showed that for learners with high CCWP, the advantage in CSL reading could still be held regardless of language distance. This finding was consistent with the empirical evidence in the brain and neuroscientific research that handwriting experience may shape the specialized neural representation and accelerate the visual processing of character formation in reading (Tan et al., 2005b; James and Atwood, 2009; Hu et al., 2018).

Another possible explanation for this is the view of mental simulation in the ECT. According to the ECT, the nature of mental activity (including metaphor interpretation, empathy, embodied learning, and language comprehension) is to re-situate by mentally simulating a virtual world with which an individual is familiar (Zwaan and Taylor, 2006; Gibjr, 2010; Gallese, 2014). Piaget (1976) put forward that L1 learning was a sensorimotor process. Given this, L2 reading acquisition may be a mental simulation that could re-situate L2 symbols in the original sensorimotor memory trace of L1 learning. As such, researchers could build correlations between memory trace and L2 through vocabulary (Henning, 1973; Lindsay and Gaskell, 2010; Macedonia, 2014), sentences (Borreggine and Kaschak, 2006; Diefenbach et al., 2013; Papesh, 2015), and even passages (Zwaan, 2004, 2016), evidenced to promote language comprehension. Therefore, as an embodied activity, Chinese character writing may bridge the L1 sensorimotor memory and L2 scripts of learners by strengthening the link between body and situation. It seems that learners good at character writing can maintain these advantages in reading. As noted in the introduction, the sociocultural theory claims that internal mediation originates from external mediation, and we may infer that for intermediate learners, internal language experience is the medium between bodily activity and external language usage in CSL learning.

### Role of Cultural Background in the Relationships Between Chinese Handwriting and Reading Comprehension

RQ3 investigated the role of cultural background in moderating the relationship between CSL character writing and reading. First, the results showed a significant effect of NSG and BSG on reading proficiency. This finding accorded with the research that intermediate learners from NSG and BSG are more proficient in reading than those from non-SG (Hsiao et al., 2015; Ke and Chan, 2017). In addition, the results further showed that CCWP could effectively distinguish between the NSG and BSG learners; that is, the NSG learners might be more proficient in reading than the BSG group. These initial results were suggestive of a relationship between cognition and context; that is, context embeds cognition and, in turn, contextual factors compose the cognition (Lakoff and Johnson, 1999; Rohrer, 2007). Even though the languages and cultures of most countries with Sinosphere cultural background have shifted, the cognitive and thinking patterns have not fallen far from the original Chinese. These results reflected those of studies (Yeh et al., 2003; Ke and Chan, 2017) that also found that readers with Chinese cultural backgrounds tend to use similar strategies in recognizing and reading characters.

Second, unlike our initial hypothesis, this study showed that CCWP × cultural background interaction effects were not statistically significant. Upon finding this result, we reconsidered whether there were any other important moderating factors at the individual level. However, there was no such factor in this study. This finding may indicate that for CSL beginners and intermediates, cultural background could not moderate the relationship between bodily activity and reading proficiency. In other words, for learners skilled in character writing, reading proficiency might fail to be constrained by non-Chinese cultural and contextual backgrounds.

One possible explanation for this finding of insignificant CCWP × cultural background interaction is differences in contexts. For the non-SG learners, because their L1s and Chinese belong to different systems, it may be difficult for them to correspond their L1s well with Chinese. Indeed, reading comprehension is a process in which the structural knowledge in the mind of a reader could interact with the text, meaning, and background of reading materials (Carrell and Eisterhold, 1983; Carrell, 1984). Also, in fact, Chinese is not a global language like English. It is possible, therefore, that CSL learners cannot wherever and whenever connect with the Chinese language in daily life. In this study, the learners of CSL at the beginning and intermediate levels might have suffered from corresponding their individual experience and knowledge to reading texts well, especially when they read texts full of unfamiliar Chinese idioms, cultures, and customs. This finding provides some explanation as to why both handwriting and cultural background are irreplaceable aspects in reading acquisition investigation.

For NSG and BSG learners, although Sinosphere countries share culture and languages, dramatic changes in languages and cultures have occurred in both China and Sinosphere countries. On the one hand, some NSG countries, such as Korea and Vietnam, have canceled character teaching and usage because of educational policy reform. On the other hand, there are some overlaps between Sinosphere languages and Chinese, but discrepancies in cultural schema, meaning, and forms of characters still exist. These discrepancies may lead to errors in CSL reading (Ellis, 2008). For example, take a shared character “
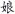
” in Japanese and Chinese as an example. In Japanese, “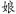,” pronounced “musume,” mainly means young girl and daughter. In Chinese, when one uses “
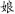
” [niang2] alone, it fundamentally means mother rather than young girl, and the meaning “daughter” disappears. In addition, characters sharing the same meaning might differ in forms, such as “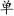,” “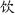,” and “
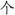
” in Chinese, or “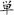,” “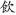,” and “
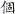
” in Japanese. An implication of this is the possibility that learners from Sinosphere countries or skilled at character writing may still meet challenges during CSL reading (Ke, 1998; Jiang, 2003; Lin and Collins, 2012; Li, 2020; Tang and Chan, 2021; Zhang and Roberts, 2021b).

Going back to the question of Alderson of whether differences in reading proficiency are a reading problem or a language problem, based on the findings in this study, we cannot simply classify reading variance into dichotomous problems, at least for CSL learners. One of the issues that emerge from previous findings is that reading may be a more complicated cognitive process than what existing research knows. Therefore, we may prefer to regard the variance of reading as a mixed cognitive problem of reading, language, and conception, different problems that possibly appear in different learning periods.

## Conclusion

Focusing on the role of CCWP in reading comprehension among CSL intermediates and adopting big data from HSK, this study contributed to a better understanding of the ECT in SLA. It revealed the embodied features of Chinese characters. First, CCWP had a positive effect on reading proficiency, which indicated an essential role of Chinese character handwriting in CSL reading. Second, language distance played a moderate role in the relationship between character writing and reading. The findings provided empirical evidence that character writing could constrain L1 negative transfer in CSL reading acquisition. Third, the cultural background of CSL learners could positively influence but might not moderate the relationship between character writing and reading proficiency.

## Implications

### Theoretical Implications

First, our study has highlighted the importance of handwriting in L2 reading. The results of this study illustrate that writing Chinese scripts by hand facilitates reading comprehension (Wang et al., 2014, 2015; Kuo et al., 2015; Tong and Yip, 2015; Tong et al., 2016; Chung et al., 2018; Zhang J. et al., 2020). In addition, the advantage of skilled character writing would remain even if L1 and L2 are linguistically distant (Sharwood Smith and Kellerman, 1986; Odlin, 1989).

Second, the findings in this study have provided a deeper insight into the cross-linguistic influence in SLA. For one thing, cross-linguistic influence occurs at the linguistic and conceptual levels (Jarvis and Pavlenko, 2008). In addition, for SLA beginners and intermediates, L1 experience may be the medium between bodily activity (i.e., character writing) and L2 usage. In turn, the L1–L2 cross-language congruity can moderate the correlations between bodily activity and L2 usage (Melby-Lervåg and Lervåg, 2011; Jeon and Yamashita, 2014). For another, language distance can be a measurement of cross-linguistic similarity or difference and may reflect the variance in L1–L2 embodied experience.

Third, this study has contributed new empirical evidence to the ECT that the effects of cultural background on investigations of CSL reading need to be reckoned with (Vygotsky, 1978; Ellis, 2008; Green and Abutalebi, 2013; Tong et al., 2016). For SLA learners in the primary stage, their L2 and the conceptual system are not completely established, and they are likely to rely on their L1 knowledge. Overall, there seems to be some evidence to indicate that although the influence of L1 cultural background on L2 scriptwriting is much less, the effect of L1 cultural background on L2 reading proficiency is more apparent.

### Practical Implications

This study also has some practical implications. First, the findings of this study suggest that it is necessary to learn the knowledge of character writing orthography at the intermediate level of CSL acquisition (Lam and McBride, 2018; Zhou et al., 2020). This finding also indicates that CSL reading acquisition develops from embodied cognition.

The findings also provide suggestions for CSL acquisition that character writing by hand may benefit from withstanding the negative transfer from L1s. Thus, it is effective for CSL learners to learn character writing in CSL reading acquisition. Additionally, for CSL character teaching, teaching materials and methods are suggested to be more diversified for learners with different L1s and cultural backgrounds.

Moreover, in CSL teaching, teachers need to pay more attention to the sociocultural backgrounds of learners. For example, having a similar background with Chinese does not mean being more proficient in character writing and orthography. Thus, it is necessary for teachers to teach script motivation (i.e., correlations between character structures, radicals, and meanings, which translated from Chinese term “
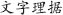
”) (Xu, 1992) of characters and keep students practicing correctly.

Finally, to help build the conceptual system of students, teachers could introduce similarities and differences in cultures, thoughts, and society between the countries of students and China, encouraging them to share their views and underlying cultural contexts (Carrell and Eisterhold, 1983).

## Limitations and Future Research

One limitation in the current study is that group-level predictor cultural background data are crudely based on the countries of residence of the participants and separated into NSG, BSG, and non-SG. As a result, case studies are needed in the future to further investigate more elaborate differences among learners with variant sociocultural backgrounds.

In addition, this study explored the role of character writing of CSL beginners and intermediates. However, this focus was limited to studying other factors that predicted reading comprehension in previous studies, including linguistic knowledge, L1 and L2 literacy experience, age of acquisition, L2 proficiency, script distance, and measurement characteristics (Koda, 2007; Jeon and Yamashita, 2014). Therefore, future research could consider these predictors and further investigate correlations among them. Also, it would be beneficial to study other latent variables (e.g., belief and anxiety) to investigate the non-linear relationship between CSL writing and reading, and how they vary across different groups^6^.

Finally, this study only explored the relationships between Chinese character handwriting and Chinese reading using the ECT at a macroscopic level. Future research could further examine the mechanism of embodiment in cognitive processing in the brain and neuroscientific technology at a microscopic level.

## Data Availability Statement

The raw data supporting the conclusions of this article will be made available by the authors, without undue reservation.

## Ethics Statement

The studies involving human participants were reviewed and approved by Institute on Educational Policy and Evaluation of International Students, Beijing Language and Culture University. Written informed consent from the participants’ legal guardian/next of kin was not required to participate in this study in accordance with the national legislation and the institutional requirements.

## Author Contributions

XC and MM conceived and designed the work. MM performed the statistical analysis and wrote the first draft of the manuscript. XC revised the manuscript critically. Both authors contributed to manuscript revision, read, and approved the submitted version.

## Conflict of Interest

The authors declare that the research was conducted in the absence of any commercial or financial relationships that could be construed as a potential conflict of interest.

## Publisher’s Note

All claims expressed in this article are solely those of the authors and do not necessarily represent those of their affiliated organizations, or those of the publisher, the editors and the reviewers. Any product that may be evaluated in this article, or claim that may be made by its manufacturer, is not guaranteed or endorsed by the publisher.

## Footnotes

1 Writing is the process of representing a language with written symbols to communicate thoughts and ideas in a readable form, while handwriting emphasize the process of scripts written by hand. We use the phrase “Chinese character writing” or “handwriting” to refer to the action of producing Chinese characters by hand from memory.

2 This study focuses on Chinese characters of Chinese writing systems. For clarity, the term “language” in this article mainly refers to written language.

3 Note the difference between the phrases “Chinese character reading” and “Chinese reading (comprehension).” The former refers to recognizing a single character without passage processing, while the latter requires readers to integrate the information of characters into words and sentences in a passage, and then understand the main idea of the passage. Chinese reading (comprehension) involves more advanced and complicated cognitive processing (e.g., understanding, memorization, interpretation, and reasoning) than character reading. This study focused on Chinese reading (comprehension).

4 The sample selected from examinees taking different HSKs in different months of the year. Scores of each HSK were comparable after equivalent processing. In addition, previous research (see Meyer, 2014) showed no significant statistical difference in the difficulty of HSKs over the years. Items of one HSK are selected from different parallel versions in pretest trials according to difficulty and discrimination, which ensures the compatibility of HSKs.

5 On the one hand, this study dichotomized CCWP into two groups to explore the between-group variance and cross-group interaction effects. On the other hand, this study grouped CCWP to keep the practical importance of results. In our study, CCWP was measured with total scores [16 points] in the HSK character writing part (one character per point). The point gap is too small to explain the variation in reality. The dichotomization of CCWP did not change the main results of this study.

6 We wish to acknowledge the reviewer who offered this advice.
